# Young Women do it Better: Sexual Dimorphism in Temporal Discrimination

**DOI:** 10.3389/fneur.2015.00160

**Published:** 2015-07-09

**Authors:** Laura Jane Williams, John S. Butler, Anna Molloy, Eavan McGovern, Ines Beiser, Okka Kimmich, Brendan Quinlivan, Sean O’Riordan, Michael Hutchinson, Richard B. Reilly

**Affiliations:** ^1^Department of Neurology, St. Vincent’s University Hospital, Dublin, Ireland; ^2^School of Medicine and Medical Science, University College Dublin, Dublin, Ireland; ^3^Trinity Centre for Bioengineering, Trinity College Dublin, Dublin, Ireland; ^4^School of Engineering, Trinity College Dublin, Dublin, Ireland; ^5^School of Medicine, Trinity College Dublin, Dublin, Ireland

**Keywords:** sexual dimorphism, temporal discrimination, movement disorders, covert attention, adult onset dystonia, superior colliculus

## Abstract

The temporal discrimination threshold (TDT) is the shortest time interval at which two sensory stimuli presented sequentially are detected as asynchronous by the observer. TDTs are known to increase with age. Having previously observed shorter thresholds in young women than in men, in this work we sought to systematically examine the effect of sex and age on temporal discrimination. The aims of this study were to examine, in a large group of men and women aged 20–65 years, the distribution of TDTs with an analysis of the individual participant’s responses, assessing the “point of subjective equality” and the “just noticeable difference” (JND). These respectively assess sensitivity and accuracy of an individual’s response. In 175 participants (88 women) aged 20–65 years, temporal discrimination was faster in women than in men under the age of 40 years by a mean of approximately 13 ms. However, age-related decline in temporal discrimination was three times faster in women so that, in the age group of 40–65 years, the female superiority was reversed. The point of subjective equality showed a similar advantage in younger women and more marked age-related decline in women than men, as the TDT. JND values declined equally in both sexes, showing no sexual dimorphism. This observed sexual dimorphism in temporal discrimination is important for both (a) future clinical research assessing disordered mid-brain covert attention in basal-ganglia disorders, and (b) understanding the biology of this sexual dimorphism which may be genetic or hormonal.

## Introduction

The temporal discrimination threshold (TDT) is the shortest time interval at which two sensory stimuli (visual, tactile, or auditory) presented sequentially are perceived as asynchronous by the observer. TDTs increase with age, being a mean of 25–30 ms <35 years of age and 35–40 ms in the 36–65 years age group (1, 2). Temporal discrimination is abnormal in disorders of basal ganglia dysfunction, including adult onset isolated focal dystonia (AOIFD) (1–9), Parkinson’s disease, and multiple systems atrophy (10–14). Temporal discrimination is proposed as a measure of the midbrain-basal ganglia network for covert attentional orienting (15) and as a meditational endophenotype in AOIFD (1, 2, 4), a condition with an increased prevalence in women (F:M ratio 2:1) (16).

Covert orienting of attention involves the “bottom-up” processing of a salient stimulus. This involves a largely involuntary attentional shift under exogenous control. Particularly salient are abrupt onset stimuli or rapid looming environmental changes. The superior colliculus (SC) and its projections, involved in multisensory detection and integration, have been implicated as key pathways in the generation of covert attentional shifts (17–19). Reflexive, covert attentional shifts, and their subsequent motor responses may have implications for survival, ranging from predator detection to navigating traffic.

During a temporal discrimination task, visual stimuli reach the wide-field sensory neurons of the SC by the extra-geniculate, retino-tectal pathway (15, 20–22), and tactile stimuli via ascending somatosensory tracts, provoking covert shifts in attention. We postulate that the level of detection of stimulus asynchrony, the TDT, is a measure of the efficacy of this short-latency midbrain network for covert attentional orienting and its ability to detect salient environmental change. As well as descending outputs from the intermediate and deep laminae of the SC to the brainstem for saccadic eye movement and the reticular formation, there are important thalamic and basal ganglia connections to the substantia nigra pars compacta and the intralaminar nuclei of the thalamus (23, 24). These basal ganglia connections from the SC provide pathways for short latency responses, allowing immediate reactions to salient environmental stimuli which may be of danger to the individual (25, 26).

In a large cohort of healthy participants, we observed age-related effects on the normal TDT; we also noted that women were faster in detecting stimulus asynchrony with significantly lower TDTs than men (1). Temporal discrimination is usually reported as a single value in milliseconds or as a corresponding *Z*-score. During TDT testing, in response to visual or tactile stimuli at varying inter-stimulus intervals, a participant reports their perception as “same” (synchronous) or “different” (asynchronous). By fitting individual participant responses to the range of inter-stimulus intervals with a cumulative Gaussian function, one may extract the mean and SD of the distribution of responses. The mean represents the point of subjective equality (PSE), the inter-stimulus interval at which participants are equally likely to respond that two stimuli are synchronous or asynchronous. The SD represents the just noticeable difference (JND), which is a measure of how sensitive participants are to changes in temporal asynchrony around the PSE. There is a strong correlation between the PSE and the TDT and also between the JND and the TDT. However, JND and PSE values are independent of each other and represent different dimensions of the TDT value (27). In this study, we aimed to examine, systematically in a large group of healthy participants, the age-related sexual dimorphism observed in temporal discrimination and in the PSE and the JND.

## Participants and Methods

### Participants

About 175 healthy participants between the ages of 20 and 65 years (88 women, mean age 41.4 years; 87 men, mean age 40.5 years) were recruited from hospital staff and visitors to the hospital. A proportion of participants were recruited for a previous study (1, 3). A full medical history was taken and participants were assessed for any evidence of neurological disorder. Exclusion criteria were a history or neurological disease, including neuropathy, visual or cognitive impairment, a history of cerebral, cervical, or brachial plexus injury, current pregnancy, and a known family history of dystonia.

### Sensory testing

**Figure 1:**
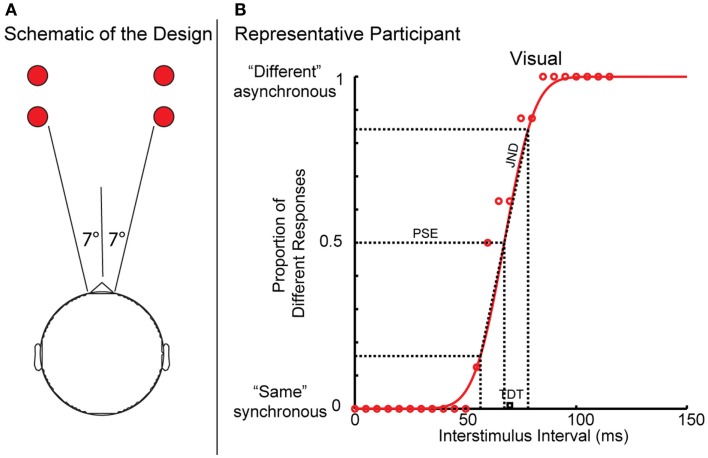
**(A,B)** Schematic of the temporal discrimination threshold experimental task and Gaussian curve of a representative participant’s data.

Visual and tactile TDT testing was carried out in a single session in a sound-proof, darkened room, as described previously (1, 3). Visual stimuli (two flashing LED lights) were positioned on a table, 7° in the participant’s peripheral visual field (Figure 1A). Tactile stimuli (non-painful electrical impulses to the index and middle fingers) were presented using square-wave stimulators (Lafayette Instruments Europe, LE12 7XT, UK) and rectangular cloth electrodes (Item # TD-141C1, Discount Disposables Post Office Box 111, St. Albans, Vermont 05478). The stimulus current was manually increased (in 0.1 mA steps) until the participant could reliably detect the stimuli. Visual or tactile stimuli, 5 ms in duration, were presented at 5 s intervals. The stimuli were initially synchronous and separation between pairs of stimuli was introduced in 5 ms steps. When the participant reported stimuli to be asynchronous on three consecutive occasions, the first of these was taken as the TDT. Visual and tactile testing was repeated four times on each side of the body (a total of 16 runs) in a random order, and the median (ms) of the four trials was used to account for a practice effect. Means of the median visual, tactile, and combined values were calculated (TDT), expressed in milliseconds. Testing was carried out by the research registrars according to a standardized protocol. Unless otherwise stated, TDT refers to combined TDT in the results and discussion.

### Analysis of point of subjective equality and just noticeable difference

The data were fitted to a cumulative Gaussian function (Figure 1B) from which the PSE and JND were extracted. The PSE represents the point at which the participant was equally likely to respond “synchronous” or “asynchronous.” The JND is the lowest point at which participant could detect a difference and this corresponds to the reliability of the responses; the higher JND the less reliable the response. In a study from our group, it was shown that the JND and PSE correlate with mean TDT values but are independent of each other, suggesting that they represent different facets of the temporal discrimination process (27).

Ethics approval for this project was obtained from the Ethics and Medical Research Committee, St. Vincent’s University Hospital. Written informed consent was obtained from all participants.

### Statistical analysis

To investigate the effect of age and sex on the TDT, regression analyses were performed. The combined TDTs for men and for women were submitted to regression analyses with age as the continuous variable. The *F* values, mean squared error (MSE), relative absolute error (RAE), coefficient of variation (CV), *R*-squared values, and corresponding *p*-values are reported along with 95% confidence intervals, *t*-values, and *p*-values for the intercept, and Beta value for the linear fit. To compare the intercept and Beta values between men and women, a regression analysis was performed on the TDT data with variables age, sex (men = 0, women = 1), and age × sex (resulting in 0 s for men and the continuous variable of age for women). The sex variable tests for differences in the intercept values between men and women. The age × sex variable tests for differences in the Beta values between men and women. The PSE and JND were submitted to the same analytical protocol.

## Results

### Temporal discrimination

**Figure 2:**
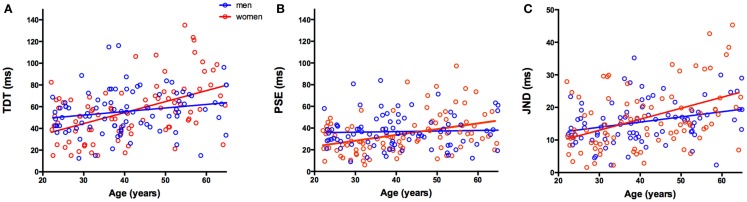
**(A–C)** Scatter plots showing relationship between age (years) and **(A)** temporal discrimination threshold (TDT) (ms), **(B)** point of subjective equality (PSE) (ms), and **(C)** just noticeable difference (JND) (ms). Red and blue dots indicate individual women (red) and men (blue) participants; red and blue lines indicate the regression fit of the data for women and men. The clear advantage in women <40 years of age in both the TDT and PSE is illustrated as well as the greater rate of decline in women, than in men, in these measures with age.

Data from all 175 participants, arranged by age and gender group for the mean TDT (in ms), mean PSE (ms), and mean JND (ms), are presented in Table 1. The TDT and age data were submitted to a linear regression analysis for both groups (women and men), and are illustrated in Figure 2.

**Table 1:** **The temporal discrimination threshold (TDT), point of subjective equality (PSE) and just noticeable difference (JND) for 175 healthy participants divided by age and sex**.

Age group	20–30 years		31–40 years		41–50 years		51–65 years	

Sex (*N*)	Women (20)	Men (20)	Women (23)	Men (27)	Women (17)	Men (17)	Women (28)	Men (23)
Mean age in years (SD)	25.2 (2.5)	25.6 (2.4)	34.3 (3.3)	35.8 (2.9)	44.9 (3.5)	43.5 (2.9)	56.5 (4.3)	56.9 (5.1)
Mean TDT in milliseconds (SD)	43.6 (20.2)	48.9 (16.3)	42.9 (17.8)	55.0 (23.9)	60.8 (26.5)	58.2 (17.4)	70.8 (29.1)	60.9 (19.5)
Mean PSE	29.5 (12.2)	34.8 (14.9)	23.6 (17.4)	36.9 (16.1)	41.7 (20.5)	38.6 (12.2)	41.5 (20.2)	37.0 (16.2)
Mean JND	11.72 (7.3)	12.8 (4.8)	15.1 (8.1)	15.0 (7.1)	16.2 (8.1)	17.5 (5.9)	22.1 (11.8)	17.3 (6.4)

*There is an effect of both age and gender on the TDT and the PSE with shorter duration TDTs and PSE in women (than men) <40 years of age. Both the TDT and the PSE increase with age in women at a greater rate (than men) so that in the 40–65 years age group, the TDT and the PSE are both longer in duration in women than in their male peers*.

### Age and gender effects in temporal discrimination threshold: Regression analysis

#### Women

For women, the analysis revealed that age explained a significant amount of the variance in the TDT values [*F* (1,86) = 27.542, MSE = 542.3, RAE = 0.89, CV = 42.0, *p* < 0.0001, *R*^2^ = 0.243, $R_{\text{ADJUSTED}}^{2}=0.234$], with a non-significant intercept 12.307, {*t*(86) = 1.432; *p* = 0.156; 95% CI [−4.7, 29.3]}, a significant Beta = 1.042, {*t*(86) = 5.248; *p* < 0.0001; 95% CI [0.647, 1.437]}(Figure 2A).

#### Men

For men, we noted a trend toward significance for age explaining the variance in the TDT values [*F* (1,85) = 3.84, MSE = 387.8, RAE = 0.97, CV = 35.3, *p* < 0.0066, *R*^2^ = 0.039, $R_{\text{ADJUSTED}}^{2}=0.028$}, with a significant intercept 42.305, {*t*(85) = 5.615; *p* < 0.0001; 95% CI [27.3, 57.3]}, and trending Beta = 0.332, {*t*(85) = 4.658 *p* < 0.0001; 95% CI [−0.022, 0.687]}.

Thus, influence of age on the mean combined TDT score is such that the TDT worsened by approximately 1 ms per annum in women (Beta = 1.042) and by 1 ms every 3 years in men (Beta = 0.332). The comparison of the fit analysis revealed a significant difference in the intercepts between the groups [*t*(173) = 2.616, *p* < 0.01], with women having a lower intercept and a significantly different Beta value [*t*(173) = 2.646, *p* < 0.01]. TDTs for 20-year-old women were approximately 13 ms faster than age-matched men, representing a 20% advantage (Figure 2A).

### Point of subjective equality

#### Women

In women, age explained a significant amount of the variance in the PSE values [*F*(1,86) = 12.997, MSE = 329.4, RAE = 0.93, CV = 53.5, *p* < 0.001, *R*^2^ = 0.131, $R_{\text{ADJUSTED}}^{2}=0.121$], with a non-significant intercept 10.844, {*t*(86) = 1.619, *p* = 0.109; 95% CI [−2.4, 24.155]}, and a significant Beta = 0.556, {*t*(86) = 3.605, *p* < 0.001; 95% CI [0.250, 0.866]}(Figure 2B).

#### Men

For men, there was no significant relationship between age and PSE values [*F*(1,85) = 0.151, MSE = 224.5, RAE = 0.998, CV = 40.7, *p* = 0.699, *R*^2^ = 0.002, $R_{\text{ADJUSTED}}^{2}=-0.01$] demonstrating a significant intercept 34.646, {*t*(85) = 6.04, *p* < 0.0001; 95% CI [23.2, 46.0]}, and a non-significant Beta = 0.053, {*t*(85) = 0.388, *p* = 0.699; 95% CI [−0.22, 0.322]}.

The comparison of fit analysis revealed a significantly different intercept between the groups [*t*(173) = −2.690, *p* < 0.01], with women having a lower intercept and significantly larger Beta values [*t*(173) = 2.44, *p* < 0.025]. Similar to the TDT analysis, the mean PSE value was lower for women than men at 20 years of age, but women were more affected by age with an increase of 1 ms in their PSE every 2 years.

### Just noticeable difference

#### Women

Age also explained a significant amount of the variance in the JND values in women [*F*(1,86) = 18.48, MSE = 83.5, RAE = 0.92, CV = 54.4, *p* < 0.001, *R*^2^ = 0.177, $R_{\text{ADJUSTED}}^{2}=0.168$], with non-significant intercept 2.952, {*t*(86) = 0.876, *p* = 0.45; 95% CI [−3.74, 9.653]} and a significant Beta = 0.335, {*t*(86) = 4.299, *p* < 0.0001; 95% CI [0.180, 0.490]}(Figure 2C).

#### Men

In men, age explained a significant amount of the variance in the JND values [*F*(1,85) = 8.35, MSE = 37.1, RAE = 0.92, CV = 39.1, *p* < 0.005, *R*^2^ = 0.089, $R_{\text{ADJUSTED}}^{2}=0.079$], with a significant intercept 9.128, {*t*(85) = 3.92, *p* < 0.001; 95% CI [4.49, 13.8]}, and a significant Beta = 0.159, {*t*(85) = 2.89, *p* < 0.005; 95% CI [0.05, 0.269]}.

The comparison of fit analysis revealed no significant difference in the intercepts between the groups [*t*(173) = −1.495, *p* = 0.137] and a trending difference in Beta values [*t*(173) = 1.817, *p* = 0.071]. Men and women had similar increases in JND values with respect to age.

## Discussion

Women, aged 20–40 years, have faster temporal discrimination than men; 20-year-old women were approximately 13 ms faster than age-matched men, representing a 20% advantage. Under the age of 40 years, women had a lower PSE value than men indicating shorter inter-stimulus intervals at which they were equally likely to detect two stimuli as synchronous or asynchronous. Women in the 20–40 years age-group were also more sensitive to change in stimulus asynchrony around their PSE as demonstrated by lower JND values. Thus, in the 20–40 years age group, women were both more sensitive and more accurate in temporal discrimination than men. Mean TDT scores in women increased (worsened) with age more than in men. Above the age of 40 years, women lose this initial advantage and had longer mean TDTs than their male peers. TDTs in women deteriorated at a rate of about 1 ms/year, while TDTs in men deteriorated at a rate of only 1 ms every 3 years. The PSE was also more influenced by age in women, remaining relatively unchanged in men despite increasing age. However, for both men and women, the JND increased as a function of age. This would suggest that the perception of asynchronous (PSE) stimuli is function of age and sex, while the reliability of the percept (JND) is a function of age but is not significantly different between men and women.

This sexual dimorphism in temporal discrimination raises interesting questions when one considers the underlying mechanisms of temporal discrimination and the pathways involved. We propose that temporal discrimination is a function of the midbrain-basal ganglia network for covert orienting of attention, with lower TDT scores representing more efficient collicular-basal ganglia processing of salient stimuli or environmental change. Numerous studies have identified cognitive, neuroanatomical, and biochemical correlates of attention and have noted sexual dimorphism (28–32). In spatial orienting tasks, such as the Posner cueing paradigm and attention networks test (ANT), women are faster than men in responding to a peripherally cued stimulus and in activating covert (exogenously controlled) attention shifts (33–35). However, women are more influenced than men by invalid cues and flankers (33, 36). There is a higher female dependency on, and preference for, visual cues (35). Electrophysiological studies also highlight increased sensitivity in women to salient visual stimuli during visual attention tasks (37).

We observed deterioration of temporal discrimination in women with age, significantly more marked than in men, indicating age-related sexual dimorphism in covert attention network function. Is the midbrain-basal ganglia network more sensitive to age in women? In attention tasks with spatial cues, reflexive allocation of attention has been found to be well preserved in older healthy individuals (38–40). Some mild differences in visual covert attention associated with aging have been identified in the more objective P1 evoked response potentials (41). The TDT task differs from traditional attention tests; however, employing much shorter inter-stimulus intervals than those used in experiments between cues and targets. Thus, it is perhaps more sensitive to deterioration with age.

Sexual dimorphism in attention strategies may be of evolutionary significance and have implications in terms of survival. Body size dimorphism can dictate sex-specific roles within species such as the need for younger females to protect offspring from predators. Female eastern gray kangaroos are physically smaller than their male counterparts and are noted to display increased vigilance behaviors and scanning for predators compared to males (42, 43).

Looming stimuli are powerful in engaging covert attention. Detection of looming stimuli with shift in attention and subsequent motor response are vital functions in terms of survival. We activate such processes while playing sport or crossing the road; in other animals, they are essential for prey and predator detection. Responses to looming stimuli are observed in many species: insects, pigeons, rodents, and non-human primates, with key involvement of the optic tectum/collicular pathways. Evidence in humans from neuroimaging studies indicates the SC is activated by multisensory looming stimuli (44, 45). Women, compared to men, underestimate the time to arrival of a visual looming stimulus, whereas with receding stimuli there is no difference between the sexes (46, 47). A looming (compared to a receding) auditory stimulus activates temporal cortical areas as well as discrete areas in the left superior posterior cerebellar cortex and a midbrain region compatible with the ascending reticular formation (48). Humans perceive looming sounds to be more salient than receding ones, with both sexes underestimating the time to arrival (49), perhaps a trait necessary to aid survival. Interestingly, women show overestimation of the spatiotemporal properties of auditory stimuli, interpreting sounds to be closer than they actually are (49). Women perceive infant cries as being closer and arriving faster compared to men’s perception; infant laughs had no such effect on anticipatory bias (50). These findings may, as with faster temporal discrimination in the young adult women, be aligned to the evolution of the female role in protecting offspring.

It is not yet clear whether the relationship between attention and sex, and its change over time, is mediated by genetic sex, or hormonal factors, or both. Interestingly, sex chromosome genes (independent of their gonadal effects) have been implicated in neurodevelopment and neural function including attention. For example the “Sex-determining region on the Y” gene (*SRY*) at Yp11.3 has been proposed to influence attention (51) through its likely modulation of dopamine biosynthesis (52, 53). Human studies have also revealed *SRY* gene expression in the thalamus, cortical areas (54), and in the substantia nigra pars compacta of male, but not female, brains within a sub-population of neurons involved in dopamine biosynthesis (55).

### Neuroanatomical gender dimorphism

Gender dimorphism has been recorded in many neuroanatomical studies, with functional magnetic resonance imaging (fMRI) revealing increased neural activation in women in areas such as the putamen, thalamus, and midbrain during visual processing (56). The midbrain-basal ganglia network has been implicated through radiological and animal model studies in the generation of covert attention (17–19, 57). Normal sexual dimorphism in the basal ganglia includes larger male putaminal volume (58); interestingly, poorer performance during a temporal discrimination task has been correlated with putaminal enlargement (4), which could be reflected in our data of higher TDTs in younger men versus women. A functional neuroimaging study in healthy participants showed that women younger than 60 years of age had 8.4% higher striatal DAT binding compared to age-matched men (59). Women have also been found to have higher striatal 18F-fluorodopa uptake than men particularly in the caudate (60). Although these studies were perhaps less sensitive due to broader age categorization and smaller numbers of participants, they do indicate superior basal ganglia network functioning in younger women versus men, as also suggested by lower TDT and PSE values in our cohort.

### Sexual dimorphism of GABAergic system

The SC plays a key role in the generation of covert attention (17–19). The wide field sensory neurons of the superficial layer of the SC (SLSC) fire (“on”) in response to visual stimuli. They then enter a “pause” phase before firing again when the stimulus is removed – the “on-pause-off” mechanism (61, 62). SLSC then relays to motor neurons of the collicular deep layers to generate a motor response. The SLSC, however, is tonically inhibited by gamma-amino-butyric-acid (GABA) outflow from the substantia nigra pars reticulata (SNPr). GABA blockade results in blurring or prolongation of the “on-pause-off response” (62), thus impairing stimulus detection and covert attention shifts. We believe reduced GABA inhibition may thus contribute to abnormal temporal discrimination (63), as short inter-stimulus gaps remain undetected and separate stimuli are perceived as synchronous. The observed increase in TDT and JND with age in both men and women may reflect a stereotyped pattern of inhibitory GABA loss within the SC-basal ganglia pathway for covert attention, with a more rapid decline in women.

Studies have also proposed that GABA is under the influence of genetic sex. X chromosome genes appear to influence enzymes involved in GABA synthesis and levels of GABA neuron markers (64). Measurement of GABA levels *in vivo* has been challenging. Magnetic resonance spectroscopy techniques have demonstrated sexual dimorphism in brain GABA levels (65, 66), albeit with conflicting results which may be explained by regional variation in concentration. A future research challenge would certainly involve assessment of both temporal discrimination and covert attentional tasks in combination with GABA measurement in areas of much smaller anatomical size, including the SC.

### Study limitations

One limitation of our study is that we assume a linear relationship of TDT with age. We have only included individuals from 20 years old up to the age of 65 years. An interesting study in the future would be to examine TDTs in both younger (children) and older individuals. It is not clear whether the observed advantage in women under the age of 40 years is secondary to genetic sex or hormonal influences. To help clarify this, a future study could also examine TDTs in relation to hormonal status and function, including menstrual variation, menarche, and menopause. However, repeated TDT testing of subjects in each menstrual cycle phase may open the experiment to a possible practice effect. The 175 healthy participants ranged in age from 20 to 65 years, and were assessed in relation to medical history and a neurological examination; however, mental state examination was not formally assessed. Subclinical cognitive impairment would, however, be highly unlikely in this cohort, and would have been evident in the reproducibility of responses in the test procedure.

### Future studies

Temporal discrimination has been applied as a mediational endophenotype in AOIFD (2) and found to be abnormal in a number of other conditions (5, 7, 10–14). Possible correlation of TDT, or covert attention and its pathways, with GABA levels in these conditions, could provide further insights into the etiopathology and would be an important area of future research.

## Conclusion

Recognition of sex differences in neural and cognitive function is vital to our understanding of neurological disorders and elucidation of pathology. Our findings add to the body of evidence that women have a superior ability to covertly shift attention. The results raise interesting questions regarding the evolutionary development of the network for covert orienting of attention in a sex- and age-dependent manner. The results also point to sexual dimorphism within the GABAergic system, which warrants further investigation. This could have clear implication for research into neurological disorders, including movement disorders, Alzheimer’s disease, schizophrenia, and autism, where GABA dysfunction has been suggested (67–72).

This work emphasizes the importance of considering both age and sex, when interpreting the TDT test and in its application as a meditational endophenotype.

## Conflict of Interest Statement

The authors declare that the research was conducted in the absence of any commercial or financial relationships that could be construed as a potential conflict of interest.
